# Geomagnetic disturbances driven by solar activity enhance total and cardiovascular mortality risk in 263 U.S. cities

**DOI:** 10.1186/s12940-019-0516-0

**Published:** 2019-09-11

**Authors:** Carolina Leticia Zilli Vieira, Danilo Alvares, Annelise Blomberg, Joel Schwartz, Brent Coull, Shaodan Huang, Petros Koutrakis

**Affiliations:** 1 Department of Environmental Health at Harvard School of Public Health, 401 Park Drive, Landmark Center 4th floor West (HSPH), 420 room, Boston, MA 02215 USA; 2 Department of Statistics, Pontifical Catholic University of Chile, Santiago, Chile; 3 Department of Biostatistics, Harvard School of Public Health, Boston, USA

**Keywords:** Geomagnetic disturbances, Health outcomes, Cardiovascular diseases, Epidemiology

## Abstract

**Background:**

Short-term geomagnetic disturbances (GMD) driven by the quasi-periodic 11-year cycle of solar activity have been linked to a broad range of adverse health effects, including cardiovascular diseases (CVD) and total deaths. We conducted a large epidemiological study in 263 U.S. cities to assess the effects of GMD on daily deaths of total, CVD, myocardial infarction (MI), and stroke.

**Methods:**

We employed a two-step meta-analysis approach, in which we estimated city-specific and season-stratified mortality risk associated with a GMD parameter (Kp index) in 263 U.S. cities. In addition, sensitivity analysis was performed to assess whether effect modification of particulate matter (PM_2.5_) in the prior day changed Kp index effects on daily deaths after adjusting for confounders.

**Results:**

We found significant association between daily GMD and total, CVD, and MI deaths. The effects were even stronger when we adjusted the models for 24-h PM_2.5_ for different seasons. For example, in the winter and fall one standard deviation of z-score Kp index increase was associated with a 0.13 and 0.31% increase in total deaths, respectively (Winter: *p* = 0.01, 95% CI: 0.02 to 0.24; Fall: *p* = 0.00001; 95% CI: 0.23 to 0.4), without adjusting for PM_2.5._ The effects of GMD on total deaths were also observed in spring and summer in the models without PM_2.5_ *(p* = 0.00001). When the models were adjusted for PM_2.5_ the total deaths increased 0.47% in winter (*p* = 0.00001, 95% CI: 0.3 to 0.65) and by 0.23% in fall (*p* = 0.001, 95% CI: 0.09 to 0.37). The effects of GMD were also significant associated with MI deaths and CVD. No positive significant association were found between Kp and stroke. The GMD effects on deaths were higher than for 24 h-PM_2.5_ alone, especially in spring and fall.

**Conclusion:**

Our results suggest that GMD is associated with total, CVD and MI deaths in 263 U. S cities. Increased mortality in the general population during GMD should be further investigated to determine whether those human physiological dynamics driven by variations in solar activity can be related to daily clinical cardiovascular observations.

**Electronic supplementary material:**

The online version of this article (10.1186/s12940-019-0516-0) contains supplementary material, which is available to authorized users.

## Introduction

Life on Earth has been long shaped by the continuous exposure to environmental electromagnetic fields. Exchange of energy between the solar wind-plasma and Earth’s magnetic field (EMF) is driven by the quasi-periodic 11-year cycle of solar activity, which generates short-term geomagnetic disturbances (GMD). GMD affect the physiology, standard metabolism and behavior pattern of humans and other species (e.g., birds, whales, reptile, insects, bacteria) [1]. Short-term GMD have been associated with a broad range of adverse health effects, including cardiovascular diseases (CVD), neurological system diseases, behavioral diseases, and total deaths [1–29].

CVD, such as myocardial infarction (MI), coronary heart diseases, and stroke, continue to be the major cause of death for all regions worldwide [30]. Heart diseases have been the first leading causes of death since 1900 in the United States, with stroke among the 50 leading causes of death every year since 1924 [30, 31]. Cornelissen et al. (2002) showed an additional risk of MI mortality of 5% during years of high solar activity compared to years of low activity in Minnesota, USA [2]. Recently, Vencloviene et al. (2014) evaluated the association between GMD and the survival of 1413 hospitalized patients with acute coronary syndromes in Lithuania [3]. Active GMD episodes during the second day after admission increased the hazard ratio by 1.58 times for cardiovascular death compared with geomagnetic quiet days [3].

Alabdulgader et al. (2018) found that not only solar wind intensity was correlated with increase in heart rate variability (HRV), but also the intensity of cosmic rays, solar radio flux, and Schumann resonance power were all associated with increased HRV and parasympathetic activity in a small health cohort in Hofuf, Saudi Arabia [28]. Sympathetic and parasympathetic nervous system activity of the autonomic nervous system (ANS) (Involuntary Nervous System) regulate functions such as the HRV, breathing, and metabolic processes in the body (U.S. National Library of Medicine, 2018). Low HRV is associated with a 32–45% increased risk of a first cardiovascular event in populations without known CVD [32], and is a predictor for sudden cardiac death that is responsible for about 25% of deaths in clinical cardiology [33].

In contrast to the positive associations discussed above Stoupel et al. (1994) observed highly significant negative correlation between daily paroxysmal atrial fibrillation (intermittent, rapid involuntary contractions of the heart muscle) and GMD activity (*r* = − 0.976, *p* = 0.02) [34]. The absolute number of daily admissions for paroxysmal atrial fibrillation was higher on geomagnetic activity I days (lower GMD activity) than level IV (higher GMD activity) days (*p* < 0.004), and stroke admissions showed the same highly significant negative correlation with increasing geomagnetic activity, but only in males of 65 years or less (*r* = − 0.99, *p* = 0.0008) [34]. Moreover, blood pressure and drastic changes in the circadian rhythm (biological 24-h circadian clock) have been reported during geomagnetic disturbances [2]. Circadian rhythm is a set of physiological and behavioral processes that exhibit a synchronized pattern with the day/night cycle periods. Shumilov et al. (2003) report that episodes of both high and extremely low levels of GMD are related to adverse effects on human health [6].

While research groups from Israel [13, 15, 24], Italy [18], Bulgaria [8–11], Mexico and Cuba [4], Canada [1], and the U.S. [2] have shown evidences of the connection between GMD and CVD deaths and other outcomes in relatively small cohorts, there is a need to determine the temporal and spatial impact of short term exposure to GMD on deaths in a large epidemiological study including CVD deaths. Consequently, we conducted a large national epidemiological study to investigate the acute effects of GMD on total and cause-specific mortality in 263 U.S. cities. In addition, we investigated the potential confounding and/or modifying effects of particulate matter with an aerodynamic diameter less than 2.5 μm (PM_2.5_) on GMD effects.

## Methods

### Population

**Fig. 1:**
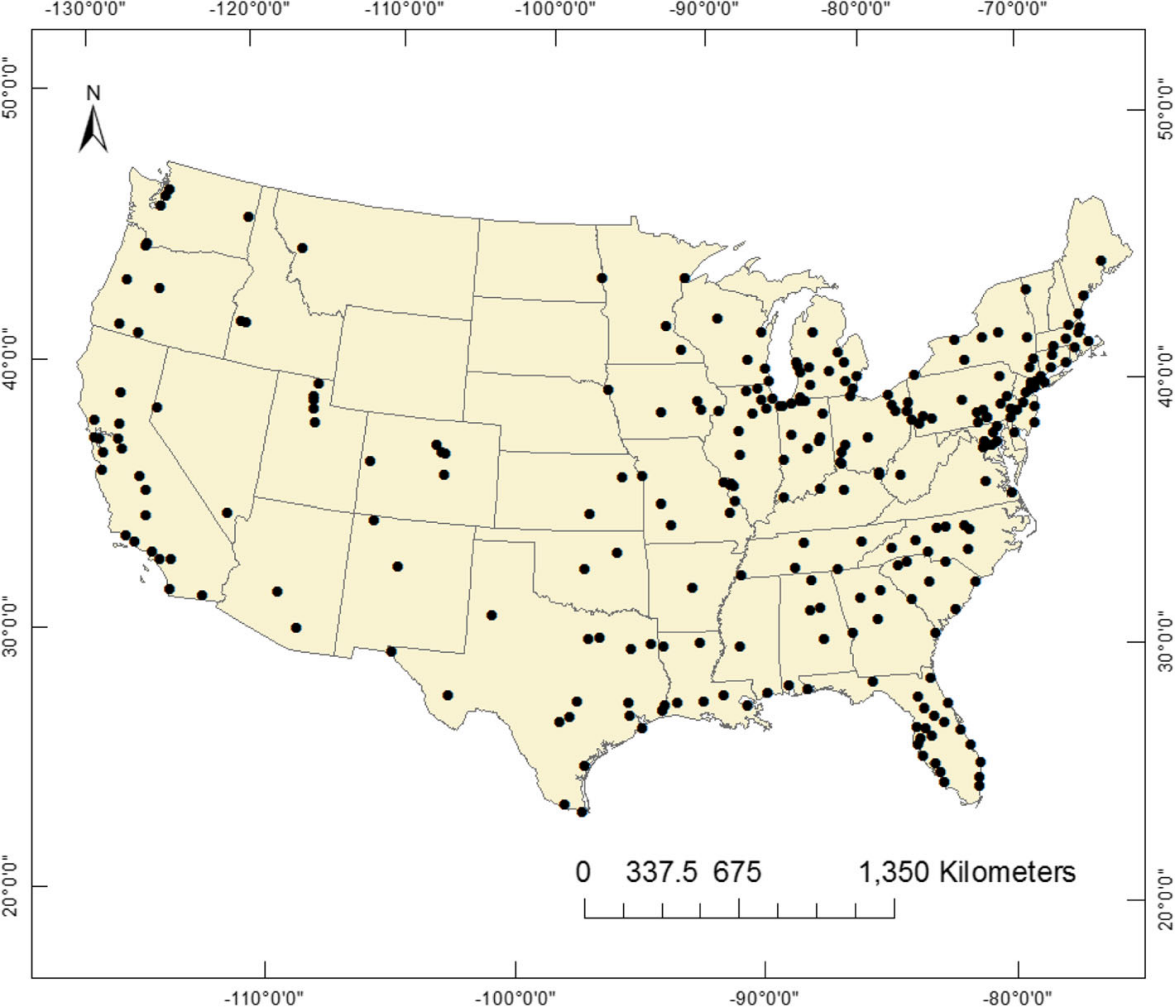
The 263 U.S. cities coordinates

Our study included daily total deaths, CVD, MI, and stroke deaths among all ages and gender from 263 U.S. cities (Fig. 1). We analyzed 2,008,990 days with 44,220,261 deaths in the study period over approximately 30 years. The mortality data up to 2006 were obtained from the National Center for Health Statistics (NCHS) website, and for years 1985 through 2013 from the Departments of Public Health for each city. The city coordinates, time-period mortality data and the total recorded deaths for each city are presented in the Additional file 1: Table S1 and Table S3). The causes of death were categorized according to the International statistical Classification codes of Diseases and Related Health Problems, Tenth Edition, as follows: all cardiovascular diseases (ICD-10th, I00-I99), stroke deaths (ICD-10th, I60–I69), MI (ICD-10th, I21-I23–8), and total non-accidental deaths. Complete description of ICD-10th causes of deaths is in the Additional file 1.

### Environmental data

**Fig. 2:**
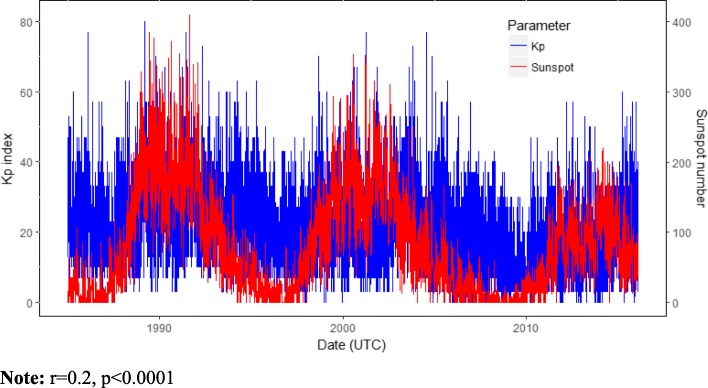
Daily sunspot number and Kp index distribution. Note: *r* 0.2, *p* < 0.0001

**Fig. 3:**
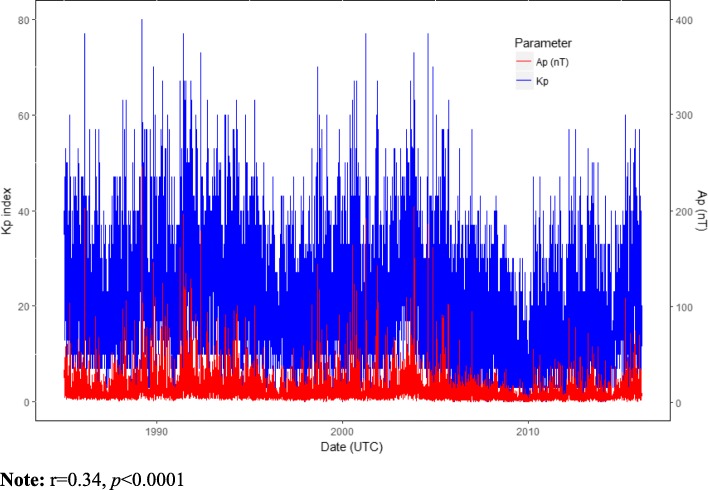
Daily Kp index and the equivalent Ap in nanoTesla (nT) distribution. **Note:** *r* 0.34, *p* < 0.0001

Daily local weather data were obtained from the National Oceanic Atmospheric Administration’s (NOAA), National Climatic Data Center. Ambient temperature and relative humidity were selected a priori based on their known associations with the outcomes or exposures [35–37]. Daily space weather data including Kp index, ap [nanoTesla (nT)] and sunspot number were downloaded from NASA Goddard Space Flight Center’s Space Physics (https://omniweb.gsfc.nasa.gov/form/dx1.html) (Figs. 2 and 3). OMNI 2 is a NASA space weather dataset recorded mostly from the ACE and WIND spacecraft located in the dayside magnetopause. High-energy GMD is directly associated with an increase in the solar activity, in which sunspot number is a parameter.

Kp index is a planetary GMD parameter generated by electric currents and the magnetic deviations on the ground (NOAA, 2019) (Fig. 3) [38]. The 3-h (h) ap (equivalent range in nT unit) index is derived from the Kp index, defined as the earliest occurring maximum 24-h value obtained by computing an 8-point running average of successive 3-h ap indices uniquely associated with the storm event (NOAA, 2019). Kp values range from 0 (no disturbance) to 9 (maximum disturbance) in 1/3 increment reported 8 times a day at 3-h intervals recorded in Coordinated Universal Time (UTC). We used daily Kp sum, which is the sum of 24 h 3-h intervals of Kp data in UTC were converted to local time for each city *i*.

Daily PM_2.5_ values for each city were calculated using a model that standardizes daily measurements for all monitors within a city boundary and prevents missing days from one monitor from adding false variability to the daily value as previously described in Zanobetti & Schwartz, 2009 [39, 40]. Acute exposure to PM_2.5_ was calculated as the mean of 24-h PM_2.5_ concentrations.

### Statistical analysis

We estimated the effects of GMD on mortality risks in 263 U.S. cities using a generalized additive models (GAM) with a quasi-Poisson link function to account for over-dispersion. The models were fitted using city-averaged variables including smooth functions (splines) with 1.5 or 2 degrees of freedom: day of the year (DOY) (1–365/366), daily ambient temperature (temp) (°C), and daily ambient relative humidity (RH) (%). Additional analyses were performed to investigate the impact of wind speed [WSPD (mph)] and planetary boundary layer height. Day of the week (DOW) (from Sunday to Saturday) and year were also included in the models. Seasons were defined as winter (December–February), spring (March–May), summer (June–August), and fall (September–November). The generic structure these models can be represented as:

1 $$ E\left({Y}_t\right)=\alpha +{\beta}_{i,s}\ast K{p}_{i,s}+{g}_{1.5}\left( DO{Y}_t\right)+{g}_2(temp)+{g}_2(RH)+{\omega}_{day,s}\ast {DOW}_t+{\lambda}_s\ast year, $$

where for each city and season, *E (Y*_*t*_*)* is the expected deaths at day *t*; α is an intercept parameter; *Kp*_*i*, *s*_ is the Kp index of the city *i* at season *s* and *β*_*i*, *s*_ its respective coefficient; *DOW*_*t*_ is a factor variable that represents the day of week at time *t* and *ω*_*day*, *s*_ is its coefficient for each factor level/day at season *s*; and *year* corresponds to the year and has *λ*_*s*_ as coefficient at season *s*. The *g*_*df*_(∙) function represents smoothing splines with *df* degrees of freedom. For a sensitivity analysis of the above model, we also included PM_2.5_ modeled through smoothing splines with 2 degrees of freedom to account for potential confounding by PM_2.5_. The z-score transformation, z = (x-μ_x_)/σ_x_, was employed for all continuous variables.

In the second stage, we used a mixed-effects meta-analysis model to estimate the overall Kp index mortality effect for each season. The model can be written as follows:

2 $$ E\left({\hat{\beta}}_{i,s}\right)={\gamma}_s+{u}_{i,s}, $$

where ${\hat{\beta}}_{i,s}$ is the estimated Kp index coefficient for city *i* at season *s* (see model 1) and *γ*_*s*_ represents the overall Kp index mortality effect (intercept) at season *s*. The city-specific random effects are represented by *u*_*i,s*_, which satisfies ${u}_{i,s}\sim N\left(0,{\sigma}_u^2\right).$

Based on model (1), the overall Kp index mortality effect at each season is calculated by:

3 $$ \mathrm{Ef}{\mathrm{f}}_{\mathrm{s}}=\exp \left(c\ast {\hat{\gamma}}_s\right), $$

where ${\hat{\gamma}}_s$ represents the Kp index estimated intercept in (2) and *c* is a predefined increment of Kp index at season *s*. Throughout this work, we consider *c* =1 as one standard deviation of z-score Kp index. Pearson correlation analysis was used to describe the relation between Kp, ap and sunspot number (Figs. 2 and 3). All analyses were performed in R software 3.4.3.

## Results

Our study included up to 2,008,990 days with data for mortality, Kp index, temperature, and relative humidity from 263 cities. The mean daily total mortality was 14 deaths/day, with winter having 16 deaths/day, spring with 14 deaths/day, summer with 13 deaths/day and fall having 14 deaths/day, including 44,220,261 deaths in the study period. The mean daily Kp index was 16.5 (~ 428 nT), with the highest seasonal average levels in spring (17.3 or ~ 758 nT) and fall (17 or ~ 758 nT) and lowest in winter and summer (15.8 or ~ 410 nT). The number of GMD storms was higher in spring and fall (Table 1). Kp index was correlated with sunspot and ap (*p* < 0.0001) (Figs. 2 and 3). Summary statistics are presented in Table 1.

**Table 1:** Summary of daily death counts and environmental parameters included in the analysis

Variable	Overall	Winter	Spring	Summer	Fall
Daily Mortality (deaths/day)	Mean (SD)				
Total	15.3 (21.5)	15.7 (23.7)	14.4 (21.4)	13.3 (20)	13.9 (20.7)
CVD	5 (8.3)	5.4 (9.3)	4.9 (8.3)	4.5 (7.6)	4.6 (7.9)
MI	1.2 (2.4)	1.4 (2.7)	1.3 (2.4)	1.1 (2.2)	1.2 (2.3)
STROKE	1 (1.6)	1.03 (1.7)	0.9 (1.6)	0.8 (1.5)	0.9 (1.5)
Environmental Parameters					
Temperature (°C)	14.2 (10.1)	3.9 (8.5)	13.6 (7.7)	23.9 (4.26)	15.07 (7.6)
*Z-score*	0 (1.0)	−1.04 (0.8)	−0.05 (0.7)	0.9 (0.4)	0.1 (0.7)
Relative Humidity (%)	66.2 (16.2)	69.4 (16.1)	69.5 (15.7)	62.1 (16.6)	65.8 (15.6)
*Z-score*	0.01 (1.0)	0.2 (1.0)	−0.25 (1.0)	0.01 (0.9)	0.1 (0.9)
Kp_index	16.5 (9.4)	15.8 (9.1)	17.3 (9.6)	15.8 (8.8)	17 (10.0)
*Z-score*	0.02 (1.0)	−0.05 (0.9)	0.1 (1.0)	−0.05 (0.9)	0.07 (1.0)
Number of GMD storms (Kp index > 5; ap index > 29)	936,368	154,316	290,004	212,180	279,868
24-h Average PM_2.5_ (μg/m^3^)	12.6 (7.5)	13.0 (7.8)	11.2 (6.2)	14.3 (8.2)	12 (7.4)
*Z-score*	0.05 (1.0)	0.13 (1.0)	−0.13 (0.8)	0.2 (1.1)	−0.04 (1.0)

**Fig. 4:**
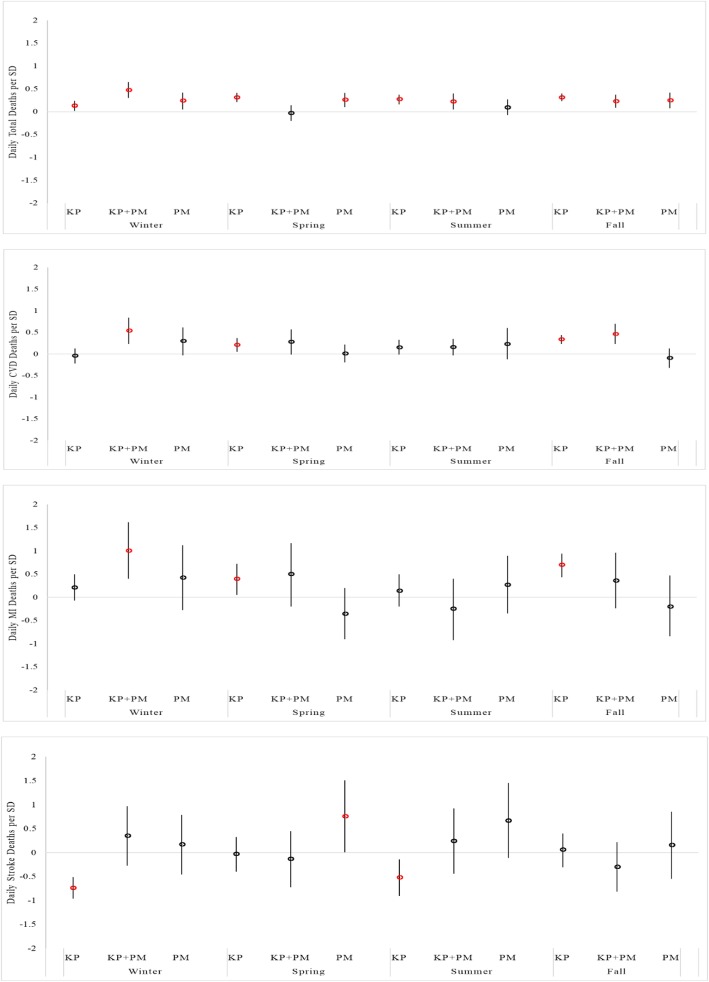
Daily city-specific and season-stratified mortality risk from the exposure to one standard deviation of z-score Kp index, Kp index adjusted for 24-h PM2.5, and only 24-h PM2.5. The models were also adjusted for daily temperature (oC), daily relative humidity (%), and day of the year (1–365/366), day of the week (DOW), year for each season (winter, spring, summer, and fall)

Table 2 presents the estimated percent increase in mortality for each standard deviation increase in z-score Kp index. Overall, we found statistically significant associations (*p* < 0.05) between daily GMD and total, CVD, and MI deaths. The results were even stronger when adjusted for 24-h average PM_2.5_ concentration in different seasons (Fig. 4). The effects of the exposure to GMD on total, CVD, and MI deaths in the day of event were similar or higher than the exposure to 24-h PM_2.5_ especially in spring and fall (Fig. 4). In winter, the association between GMD and total, CVD and MI deaths were significantly larger in the models adjusted for PM_2.5._ No significant associations were observed between 24 h-PM_2.5_ and CVD and MI alone (Fig. 4).

**Table 2:** Estimated daily ***percent*** increase in mortality (95% CI) associated with one standard deviation of z-score Kp index

	Total	CVD	MI	Stroke
**Winter**	0.13 (0.02,0.24)*	−0.04(− 0.22,0.13)	0.21(− 0.07,0.5)	−0.74(− 0.96,-0.51)****
Spring	0.31 (0.21,0.41)****	0.21 (0.05,0.37)**	0.4 (0.05,0.72)*	−0.03(− 0.4,0.33)
Summer	0.27 (0.16,0.37)****	0.15(−0.01,0.33)	0.14(−0.2,0.5)	−0.52(− 0.14,-0.9)**
Fall	0.31 (0.23,0.4)****	0.34 (0.23,0.44)****	0.7 (0.43,0.94)****	0.06(−0.3,0.4)

*p-value < 0.05; **p < 0.001; ***p < 0.0001; ****p < 0.00001

We found that an increase of 0.13% in total mortality in winter (*p* = 0.01; 95% CI: 0.02 to 0.24), 0.31% in spring (*p* = 0.00001; 95% CI: 0.21 to 0.41), 0.27% in summer (*p* = 0.00001; 95% CI: 0.16 to 0.37), and 0.31% increase in total deaths in fall season (*p* = 0.00001; 95% CI: 0.23 to 0.4) for one standard deviation increase in z-score Kp index in the models without adjusting for PM_2.5_. When adjusted for PM_2.5_, total deaths increased by 0.47% for each standard deviation of z-score Kp index increase in winter (*p* = 0.00001; 95% CI: 0.3 to 0.65), 0.22% in summer (*p* = 0.00001; 95% CI: 0.05 to 0.4), and 0.23% in fall (*p* = 0.001; 95% CI: 0.09 to 0.37). No statistically significant associations were found between GMD and total deaths in spring in the models adjusted for PM_2.5_ (Table 3).

**Table 3:** Estimated ***percent*** increase in mortality (95% CI) associated with one standard deviation of z-score Kp index adjusted for PM_2.5_

	Total	CVD	MI	Stroke
Winter	0.47 (0.3,0.65)****	0.54 (0.23,0.84)***	1.0 (0.4,1.62)***	0.35(− 0.27,0.97)
Spring	−0.03(− 0.2,0.14)	0.28(− 0.01,0.57)	0.5(− 0.2,1.17)	−0.13(− 0.72,0.45)
Summer	0.22 (0.05,0.4)**	0.16(−0.03,0.35)	−0.25(− 0.92,0.4)	0.24(− 0.44,0.92)
Fall	0.23 (0.09,0.37)**	0.46 (0.23,0.7)****	0.36(−0.24,0.96)	−0.3(− 0.81,0.22)

*p-value < 0.05; **p < 0.001; ***p < 0.0001; ****p < 0.00001

The analysis of the effects of GMD on CVD and MI deaths showed statistically significant associations in spring and fall seasons in the models without adjusting for PM_2.5._ CVD mortality increased by 0.21% for one standard deviation of z-score Kp index increase in spring (*p* = 0.008; 95% CI: 0.05 to 0.37), and by 0.34% in fall (*p* = 0.00001; 95% CI: 0.23 to 0.44). In the models adjusted for PM_2.5_, the CVD deaths increased by 0.54% in winter (*p* = 0.0005; 95% CI: 0.23 to 0.84) and by 0.46% in fall (*p* = 0.00001; 95% CI: 0.23 to 0.7) for one standard deviation of z-score Kp index increase. No statistically significant associations were found in spring and summer seasons in the models adjusted for PM_2.5_ (Table 3).

One standard deviation of z-score Kp index increased MI deaths by 0.4% in spring (*p* = 0.02; 95% CI: 0.05 to 0.72) and by 0.7% in fall (*p* = 0.00001; 95% CI: 0.43 to 0.94), without adjusting for PM_2.5_. When the models were adjusted for PM_2.5_, MI deaths increased 1.0% for each standard deviation of z-score Kp index in winter (*p* = 0.0001; 95% CI: 0.4 to 1.62). No significant associations were found in other seasons (Table 3). For stroke deaths, GMD was negatively significantly associated in winter, spring and summer (Table 2). No significant associations were found between GMD and stroke after adjusting for PM_2.5_ (Table 3). The inclusion of WSPD and HPBL in the models had no effect on the associations found between GMD and mortality rates.

## Discussion

Overall, this study suggests that GMD increases city-specific and season-stratified total, CVD, and MI deaths in the selected 263 U.S. cities (Fig. 2). The effects were stronger when we adjusted the models for PM_2.5_ for different seasons. In these models, the associations between GMD and CVD and MI were only statistically significant in winter_,_ indicating that both predictors are important for this season. Previous epidemiological studies described higher incidence of CVD mortality in winter in elderly patients [41, 42]. In addition, while numerous studies of environmental exposure risk have described the seasonal impact of PM_2.5_ on mortality rates [43], this study shows that short-term effects of GMD on total, CVD, and MI deaths were similar to or stronger than the effects of 24 h-PM_2.5_ exposures_._

The effects of GMD on total deaths were found in all seasons, and on CVD and MI deaths in spring and fall. Also in spring and fall, the effects of GMD on total, CVD, and MI deaths were higher than for PM_2.5_ alone. Seasonal short-term GMD variability is notably stronger during equinoctial seasons [44, 45]. Major GMD, also known as geomagnetic storms, occur when variations in the solar wind driven by solar activity transfer energy from the solar wind into Earth’s magnetosphere. Earth’s magnetosphere is a highly dynamic area around the planet that responds dramatically to solar variations by producing changes in the radiation belts, changes in the ionosphere, and in the environmental electric currents (NOAA, 2018). Diurnal earth magnetic field variations can range between few tens of nT to 100–500 nT over a 72 h period. Increased equinoctial GMD may explain the higher impact of GMD on mortality rates in spring and fall.

The direct impact of environmental electric and magnetic fields produced during GMD [46] on the human ANS may explain the effects of GMD on total, CVD, and MI deaths found in our study. Interactions between GMD with ANS are likely to induce a cascade of reactions in the body’s electrophysiology that culminate in the collapse of organ functions and death. Studies have described the mechanisms by which GMD may regulate ANS and body systems via a magneto-reception system [47]. Magneto-reception is a sense which allows living beings to detect EMF variations. EMF may trigger quantum chemical reactions in photosensitive retina proteins called cryptochromes and/or within magneto-receptors in cells containing magnetite (magnetic mineral iron) that activate specific structures of the central nervous system, such as the suprachiasmatic nucleus and the ANS [47]. The overlap between diurnal cycle of solar radiation and episodes of GMD on photo/magnetoreceptors may over-stimulate functioning of the central nervous system and disrupt the standard 24-h circadian rhythm processes, playing a dramatic role in the regulation of cardiovascular physiology and other systems [48]. The close alignment between the geomagnetic activity rhythms and the electrophysiology of the human body has been observed in ultrasound waves from heart structures [echocardiogram (ECG)], in brain waves (electroencephalogram [EEG)], and peripheral nerve activity that is controlled by the ANS [1, 2, 4].

In this context, increased GMD is likely to activate in humans: (1) the photo/magneto-reception system, including the ferromagnetic receptors and the cryptochrome protein [14–16, 27]; (2) cell membrane excitability (thermal noise), by the enhancement of ionic motion, signaling, and accumulation in ion channels, with primary focus on intracellular Ca^+ 2^; (3) the surface charges and small electric currents (as electric fields) among cells with capacity to distort the membrane shape; (4) the chemical bonds via radical-pair-reaction; (5) the production of reactive oxygen species (ROS) or reactive nitrogen species (RNS), inducing acute and chronic episodes of oxidative stress that contribute to many pathological conditions; (6) regulation of melatonin secretion and 24-h circadian rhythm disruption [14–16, 48–50]. These processes appear to be mutually dependent.

Ca^2+^ regulates many aspects of cell function, including energy metabolism, signal transduction, hormonal regulation, cellular motility, and apoptosis [51, 52]. In cardiac cells, the GMD-photo/magnetoreceptors-ANS may induce prolonged cardiac action potential (change in voltage across cardiac cell membrane), activating intracellular Ca^2+^ overload through voltage-gated Ca^2+^ channels. Increased intracellular Ca^2+^ accumulation activates the calcium-sensor protein called calmodulin that regulates the e.g. plasma membrane Ca^2+^ pump, intracellular Ca^2+^ dependent proteins, and enzyme expression including adenylyl cyclase (AC) [53]. AC produce cyclic adenosine monophosphate (cAMP), which controls protein kinase A (PKA) activity [54]. cAMP is a second messenger that regulates the function of ion channels across the cell membranes. The activity of AC-cAMP/PKA generates spontaneous action potential [54]. Dysregulation of ANS and increased expression of AC-cAMP/PKA lead to cardiac dysrhythmias and arrhythmias [53, 54], which have been interrelated with several abnormal arrhythmia-related conditions including acute myocardial infarction, congestive heart failure, diabetic neuropathy and sudden cardiac death [55–57]. Patients with congestive heart failure have high incidence of sudden cardiac death attributed to lethal cardiac arrhythmias [57]. A recent study found that ANS activity presents strongly synchronized patterns with the time-varying magnetic fields associated with the geomagnetic field and Schumann resonances (atmospheric electric fields) in a small healthy cohort [29]. The same group demonstrated that GMD was strongly correlated with increased parasympathetic activity and HRV [28].

Previous epidemiological studies have found significant associations between GMD and both MI and CVD deaths [2–4, 20]. In Lithuania, Vencloviene et al. (2014) showed that high GMD during the second day after admission increased the hazard ratio by 1.58 times for cardiovascular death compared with quiet days [3]. Patients with impaired cardiovascular system functions demonstrated deterioration in capillary blood flow during high GMD [3]. They also observed that increased GMD may modify the association between short-term nitrogen dioxide (NO_2_) exposure and emergency hospitalization for acute coronary syndrome (ACS) [3]. Mendonza et al. (2004) observed that MI events were statistically significantly associated with GMD (ap > 50 nT) in five large hospitals at Havana [21]. Moreover, Stoupel et al. (1994) found stroke deaths were negatively significantly associated with GMD [34]. These results are similar with our findings.

Studies have suggested that animal and human standard physiology are synchronized with EMF, being highly sensitive to its unpredictable GMD oscillations. The deep penetration of earth magnetic field into living tissues and cells added to the increase of atmospheric electric field variations during GMD may deeply modify the circadian rhythm processes and recovery properties of human body, leading to its collapse and death. Future studies are needed to quantify the synchronization between diurnal and nocturnal EMF with human electrophysiology during different intensities of GMD periods, which may be related to daily clinical cardiovascular observations. The comprehension of the cyclic impact of natural environmental risk factors driven by solar activity on human health is fundamental to understand evolutionary and adaptive aspects of life on Earth.

## Conclusion

Overall, this study suggests that GMD increase daily total, CVD, and MI deaths, even fitting models with 24-h PM_2.5_. The GMD effects on deaths were higher than for PM_2.5_ alone especially in spring and fall. Increased mortality in the general population during GMD should be further investigated to determine whether those human physiological dynamics driven by variations in solar activity can be related to daily clinical cardiovascular observations.

## Additional file

Additional file 1: **Table S1.** ICD-10th edition classification for all causes of deaths. **Table S1a.** ICD-10th edition classification for diseases of the circulatory system. **Table S2.** City coordinates and time-period mortality data. **Table S3.** City-recorded deaths (DOCX 107 kb)

## Data Availability

The datasets used and/or analyzed during the current study are available from the corresponding author on reasonable request.
