# Supplementary material for: Geomagnetic disturbances driven by solar activity enhance total and cardiovascular mortality risk in 263 U.S. cities
Source: Environ Health. 2019 Sep 11;18:83. doi: 10.1186/s12940-019-0516-0 (PMC6739933; doi:10.1186/s12940-019-0516-0)
Supplement: Supplementary file 1 — Table S1. ICD-10th edition classification for all causes of deaths. Table S1a. ICD-10th edition classification for diseases of the circulatory system. Table S2. City coordinates and time-period mortality data. Table S3. City-recorded deaths (DOCX 107 kb) [file 12940_2019_516_MOESM1_ESM.docx]

**Additional file 1**

**Geomagnetic Disturbances Driven by Solar Activity Enhance Total and Cardiovascular Mortality Risk in 263 U.S. Cities**

**Carolina Leticia Zilli Vieira^1*^, Danilo Alvares^2^, Annelise Blomberg^1^, Joel Schwartz^1^, Brent Coull^3^, Shaodan Huang^1^, Petros Koutrakis^1^.**

^1^Department of Environmental Health at Harvard School of Public Health

^2^Department of Statistics at Pontifical Catholic University of Chile

^3^Department of Biostatistics at Harvard School of Public Health

Table S1. ICD-10^th^ edition classification for all causes of deaths

| **ICD- 10th** |  |  |
| --- | --- | --- |
| **Chapter** | **Blocks** | **Title** |
| **I** | **A00–B99** | Certain infectious and parasitic diseases |
| **II** | **C00–D48** | Neoplasms |
| **III** | **D50–D89** | Diseases of the blood and blood-forming organs |
| **IV** | **E00–E90** | Endocrine, nutritional and metabolic diseases |
| **V** | **F00–F99** | Mental and behavioural disorders |
| **VI** | **G00–G99** | Diseases of the nervous system |
| **VII** | **H00–H59** | Diseases of the eye and adnexa |
| **VIII** | **H60–H95** | Diseases of the ear and mastoid process |
| **IX** | **I00–I99** | Diseases of the circulatory system |
| **X** | **J00–J99** | Diseases of the respiratory system |
| **XI** | **K00–K93** | Diseases of the digestive system |
| **XII** | **L00–L99** | Diseases of the skin and subcutaneous tissue |
| **XIII** | **M00–M99** | Diseases of the musculoskeletal system and connective tissue |
| **XIV** | **N00–N99** | Diseases of the genitourinary system |
| **XV** | **O00–O99** | Pregnancy, childbirth and the puerperium |
| **XVI** | **P00–P96** | Certain conditions originating in the perinatal period |
| **XVII** | **Q00–Q99** | Congenital malformations, deformations and chromosomal abnormalities |
| **XVIII** | **R00–R99** | Symptoms, signs and abnormal clinical and laboratory findings |
| **XIX** | **S00–T98** | Injury, poisoning and certain other consequences of external causes |
| **XX** | **V01–Y98** | External causes of morbidity and mortality |
| **XXI** | **Z00–Z99** | Factors influencing health status and contact with health services |
| **XXII** | **U00–U99** | Codes for special purposes |

Table S1a. ICD-10^th^ edition classification for diseases of the circulatory system

| **I00-I02** | **Acute rheumatic fever** |
| --- | --- |
| **I05-I09** | **Chronic rheumatic heart diseases** |
| **I10-I15** | **Hypertensive diseases** |
| **I20-I25** | **Ischaemic heart diseases** |
| **I26-I28** | **Pulmonary heart disease and diseases of pulmonary circulation** |
| **I30-I52** | **Other forms of heart disease** |
| **I60-I69** | **Cerebrovascular diseases** |
| **I70-I79** | **Diseases of arteries, arterioles and capillaries** |
| **I80-I89** | **Diseases of veins, lymphatic vessels and lymph nodes, not elsewhere classified** |
| **I95-I99** | **Other and unspecified disorders of the circulatory system** |

Table S2. City coordinates and time-period mortality data

|  |  |  |  |  |  |
| --- | --- | --- | --- | --- | --- |
|  |  |  |  | **Time-period mortality** | |
| **CITY_NAME** | **State** | **Latitude** | **Longitude** | Start_date | End_date |
| **Akron** | OH | 41.068 | -81.522 | 1/1/1985 | 12/31/2006 |
| **Albany** | NY | 42.658 | -73.767 | 1/1/1985 | 12/31/2009 |
| **Albuquerque** | NM | 35.103 | -106.602 | 1/1/1985 | 12/31/2009 |
| **Allentown** | PA | 40.600 | -75.484 | 1/1/1985 | 12/31/2009 |
| **Alpine** | TX | 30.263 | -103.654 | 1/3/1985 | 12/20/2009 |
| **Anaheim** | CA | 33.836 | -117.881 | 1/1/1985 | 12/31/2008 |
| **Ann Arbor** | MI | 42.276 | -83.727 | 1/1/1985 | 12/31/2009 |
| **Annandale** | VA | 38.831 | -77.214 | 1/1/1985 | 12/31/2009 |
| **Annapolis** | MD | 38.952 | -76.491 | 1/1/1985 | 12/31/2009 |
| **Asheville** | NC | 35.591 | -82.548 | 1/1/1985 | 12/31/2009 |
| **Ashland** | KY | 38.461 | -82.645 | 1/1/1985 | 12/31/2009 |
| **Athens** | GA | 33.965 | -83.390 | 1/1/1985 | 12/31/2011 |
| **Atlanta** | GA | 33.795 | -84.387 | 1/1/1985 | 12/31/2011 |
| **Atlantic City** | NJ | 39.366 | -74.432 | 1/1/1985 | 12/31/2008 |
| **Augusta** | GA | 33.467 | -81.993 | 1/1/1985 | 12/31/2011 |
| **Aurora** | CO | 39.696 | -104.791 | 1/1/1985 | 12/31/2009 |
| **Austin** | TX | 30.308 | -97.763 | 1/1/1985 | 5/23/1999 |
| **Aztec** | NM | 36.820 | -108.011 | 1/1/1985 | 12/31/2009 |
| **Bakersfield** | CA | 35.351 | -119.028 | 1/1/1985 | 12/31/2008 |
| **Baltimore** | MD | 39.312 | -76.619 | 1/1/1985 | 12/31/2009 |
| **Bangor** | ME | 44.824 | -68.792 | 1/1/1985 | 12/31/2009 |
| **Bath** | NY | 42.337 | -77.318 | 1/1/1985 | 12/31/2009 |
| **Baton Rouge** | LA | 30.444 | -91.098 | 1/1/1985 | 12/31/2006 |
| **Beaver Dam** | WI | 43.462 | -88.841 | 1/1/1985 | 12/30/2006 |
| **Bel Air** | MD | 39.535 | -76.336 | 1/1/1985 | 12/31/2009 |
| **Bend** | OR | 44.058 | -121.296 | 1/1/1985 | 12/31/2009 |
| **Birmingham** | AL | 33.523 | -86.826 | 1/1/1985 | 12/31/2006 |
| **Boise** | ID | 43.611 | -116.232 | 1/1/1985 | 12/31/2000 |
| **Boston** | MA | 42.354 | -71.069 | 1/1/1985 | 12/31/2013 |
| **Boulder** | CO | 40.024 | -105.254 | 1/1/1985 | 12/31/2009 |
| **Brownsville** | TX | 25.928 | -97.489 | 1/1/1985 | 12/31/2009 |
| **Burlington** | VT | 44.484 | -73.220 | 1/2/1985 | 12/31/2009 |
| **Canton** | OH | 40.813 | -81.377 | 1/1/1985 | 12/31/2006 |
| **Cedar Rapids** | IA | 41.982 | -91.668 | 1/1/1985 | 12/31/2006 |
| **Charleston** | SC | 32.835 | -80.010 | 1/1/1985 | 12/31/2011 |
| **Charleston** | WV | 38.350 | -81.633 | 1/1/1985 | 12/31/2006 |
| **Charlotte** | NC | 35.202 | -80.832 | 1/1/1985 | 12/31/2009 |
| **Chattanooga** | TN | 35.036 | -85.277 | 1/1/1985 | 12/31/2006 |
| **Chesterbrook** | PA | 40.075 | -75.459 | 1/1/1985 | 12/31/2009 |
| **Chesterfield** | SC | 34.728 | -80.096 | 1/3/1985 | 12/31/2011 |
| **Chicago** | IL | 41.872 | -87.669 | 1/1/1985 | 12/31/2006 |
| **Chico** | CA | 39.730 | -121.816 | 1/1/1985 | 12/31/2008 |
| **Cincinnati** | OH | 39.144 | -84.512 | 1/1/1985 | 12/31/2006 |
| **Cleveland** | OH | 41.489 | -81.652 | 1/1/1985 | 12/31/2006 |
| **Colorado Springs** | CO | 38.846 | -104.741 | 1/1/1985 | 12/31/2009 |
| **Columbia** | SC | 34.031 | -81.018 | 1/1/1985 | 12/31/2011 |
| **Columbus** | GA | 32.482 | -84.947 | 1/1/1985 | 12/31/2011 |
| **Columbus** | OH | 39.981 | -82.989 | 1/1/1985 | 12/31/2006 |
| **Conroe** | TX | 30.242 | -95.458 | 1/1/1985 | 12/31/2009 |
| **Corpus Christi** | TX | 27.756 | -97.414 | 1/1/1985 | 12/31/2009 |
| **Covington** | KY | 39.071 | -84.521 | 1/1/1985 | 12/31/2009 |
| **Dade City** | FL | 28.364 | -82.195 | 1/1/1985 | 12/31/2006 |
| **Dallas** | TX | 32.801 | -96.791 | 1/1/1985 | 12/31/2009 |
| **Davenport** | IA | 41.546 | -90.586 | 1/1/1985 | 12/31/2006 |
| **Dayton** | OH | 39.762 | -84.197 | 1/1/1985 | 12/31/2006 |
| **Daytona Beach** | FL | 29.212 | -81.023 | 1/1/1985 | 12/31/2006 |
| **Denver** | CO | 39.718 | -104.962 | 1/1/1985 | 12/31/2009 |
| **Des Moines** | IA | 41.588 | -93.632 | 1/1/1985 | 12/31/2006 |
| **Detroit** | MI | 42.368 | -83.106 | 1/1/1985 | 12/31/2009 |
| **Dover** | DE | 39.157 | -75.536 | 1/1/1985 | 12/31/2006 |
| **Duluth** | MN | 46.822 | -92.081 | 1/1/1985 | 12/31/2009 |
| **Durham** | NC | 36.013 | -78.909 | 1/1/1985 | 12/31/2009 |
| **Dutchess** | NY | 41.778 | -73.747 | 1/1/1985 | 12/31/2009 |
| **East Saint Louis** | IL | 38.599 | -90.074 | 1/1/1985 | 12/31/2006 |
| **El Centro** | CA | 32.789 | -115.567 | 1/1/1985 | 12/30/2008 |
| **El Paso** | TX | 31.805 | -106.435 | 1/1/1985 | 12/31/2009 |
| **Elizabeth** | NJ | 40.664 | -74.211 | 1/1/1985 | 12/31/2008 |
| **Elkhart** | IN | 41.678 | -85.969 | 1/1/1985 | 12/31/2008 |
| **Erie** | PA | 42.108 | -80.081 | 1/1/1985 | 12/31/2009 |
| **Eugene** | OR | 44.055 | -123.113 | 1/1/1985 | 12/31/2009 |
| **Evansville** | IN | 37.993 | -87.553 | 1/1/1985 | 12/31/2008 |
| **Everett** | WA | 47.943 | -122.197 | 1/1/1985 | 12/31/2008 |
| **Fargo** | ND | 46.856 | -96.812 | 1/1/1985 | 12/31/2009 |
| **Fayetteville** | NC | 35.054 | -78.944 | 1/1/1985 | 12/31/2009 |
| **Flint** | MI | 43.015 | -83.692 | 1/1/1985 | 12/31/2009 |
| **Fort Lauderdale** | FL | 26.120 | -80.199 | 1/1/1985 | 12/31/2006 |
| **Fort Myers** | FL | 26.593 | -81.852 | 1/1/1985 | 12/31/2006 |
| **Fort Pierce** | FL | 27.445 | -80.357 | 1/1/1985 | 12/31/2006 |
| **Fort Wayne** | IN | 41.084 | -85.138 | 1/1/1985 | 12/31/2008 |
| **Fort Worth** | TX | 32.746 | -97.318 | 1/1/1985 | 12/31/2009 |
| **Fredericktown** | MO | 37.559 | -90.294 | 1/2/1985 | 5/29/1985 |
| **Fresno** | CA | 36.783 | -119.786 | 1/1/1985 | 12/31/2006 |
| **Gainesville** | FL | 29.659 | -82.360 | 1/1/1985 | 12/31/2006 |
| **Galveston** | TX | 29.268 | -94.846 | 1/1/1985 | 12/31/2003 |
| **Gary** | IN | 41.578 | -87.343 | 1/1/1985 | 12/31/2006 |
| **Geneva** | IL | 41.886 | -88.311 | 1/1/1985 | 12/31/2006 |
| **Gettysburg** | PA | 39.832 | -77.222 | 1/2/1985 | 12/31/2006 |
| **Grand Haven** | MI | 43.038 | -86.191 | 1/1/1985 | 12/31/2006 |
| **Grand Junction** | CO | 39.086 | -108.567 | 1/1/1985 | 12/31/2006 |
| **Grand Rapids** | MI | 42.956 | -85.621 | 1/1/1985 | 12/31/2006 |
| **Green Bay** | WI | 44.513 | -88.002 | 1/1/1985 | 12/31/2006 |
| **Greensboro** | NC | 36.075 | -79.826 | 1/1/1985 | 12/31/2006 |
| **Greensburg** | PA | 40.307 | -79.542 | 1/1/1985 | 12/31/2006 |
| **Greenville** | SC | 34.848 | -82.387 | 1/1/1985 | 12/31/2011 |
| **Hartford** | CT | 41.767 | -72.687 | 1/1/1985 | 12/31/2006 |
| **Hickory** | NC | 35.723 | -81.345 | 1/1/1985 | 12/31/2006 |
| **Holland** | MI | 42.791 | -86.129 | 1/1/1985 | 12/31/2006 |
| **Houston** | TX | 29.776 | -95.416 | 1/1/1985 | 12/31/2006 |
| **Huntsville** | AL | 34.711 | -86.613 | 1/1/1985 | 12/31/2006 |
| **Indianapolis** | IN | 39.789 | -86.141 | 1/1/1985 | 12/31/2006 |
| **Iowa City** | IA | 41.655 | -91.531 | 1/1/1985 | 12/31/2006 |
| **Ironton** | OH | 38.529 | -82.665 | 1/4/1985 | 12/25/2009 |
| **Jackson** | MS | 32.318 | -90.198 | 1/1/1985 | 12/31/2006 |
| **Jacksonville** | FL | 30.295 | -81.653 | 1/1/1985 | 12/31/2006 |
| **Jersey City** | NJ | 40.726 | -74.062 | 1/1/1985 | 12/31/2006 |
| **Joliet** | IL | 41.526 | -88.065 | 1/1/1985 | 12/31/2006 |
| **Kalamazoo** | MI | 42.264 | -85.610 | 1/1/1985 | 12/31/2006 |
| **Kansas City** | KS | 39.111 | -94.685 | 1/1/1985 | 12/31/2006 |
| **Kenosha** | WI | 42.582 | -87.852 | 1/1/1985 | 12/31/2006 |
| **Klamath Falls** | OR | 42.192 | -121.724 | 1/1/1985 | 12/30/2006 |
| **Knoxville** | TN | 35.990 | -83.953 | 1/1/1985 | 12/31/2006 |
| **La Porte** | IN | 41.599 | -86.708 | 1/1/1985 | 12/31/2006 |
| **Lafayette** | IN | 40.415 | -86.874 | 1/2/1985 | 12/31/2006 |
| **Lafayette** | LA | 30.214 | -92.033 | 1/1/1985 | 12/31/2006 |
| **Lake Charles** | LA | 30.240 | -93.207 | 1/1/1985 | 12/31/2006 |
| **Lake City** | MI | 44.335 | -85.215 | 1/5/1985 | 12/27/2006 |
| **Lakeland** | FL | 28.062 | -81.959 | 1/1/1985 | 12/31/2006 |
| **Lansing** | MI | 42.727 | -84.566 | 1/1/1985 | 12/31/2006 |
| **Las Vegas** | NV | 36.148 | -115.174 | 1/1/1985 | 12/31/2006 |
| **Lawrenceburg** | TN | 35.251 | -87.353 | 1/1/1985 | 12/31/2006 |
| **Layton** | UT | 41.086 | -111.949 | 1/1/1985 | 12/31/2006 |
| **Lexington** | KY | 38.028 | -84.499 | 1/1/1985 | 12/31/2006 |
| **Lisbon** | OH | 40.759 | -80.759 | 1/1/1985 | 4/4/2011 |
| **Little Rock** | AR | 34.734 | -92.355 | 1/1/1985 | 12/29/2006 |
| **Logan** | UT | 41.747 | -111.823 | 1/1/1985 | 12/31/2006 |
| **Los Angeles** | CA | 34.042 | -118.305 | 1/1/1985 | 12/31/2006 |
| **Louisville** | KY | 38.223 | -85.731 | 1/1/1985 | 12/31/2006 |
| **Lubbock** | TX | 33.564 | -101.881 | 1/1/1985 | 12/31/2006 |
| **Macon** | GA | 32.874 | -83.675 | 1/1/1985 | 12/31/2011 |
| **Madison** | IL | 38.681 | -90.157 | 1/1/1985 | 12/31/2006 |
| **Marshall** | TX | 32.534 | -94.362 | 1/1/1985 | 12/30/2006 |
| **McAllen** | TX | 26.216 | -98.239 | 1/1/1985 | 12/31/2006 |
| **Medford** | OR | 42.336 | -122.840 | 1/1/1985 | 12/31/2006 |
| **Medford** | WI | 45.151 | -90.350 | 1/3/1985 | 12/31/2006 |
| **Melbourne** | FL | 28.138 | -80.652 | 1/1/1985 | 12/31/2006 |
| **Melville** | NY | 40.795 | -73.403 | 1/1/1985 | 12/31/2006 |
| **Memphis** | TN | 35.122 | -89.960 | 1/1/1985 | 12/31/2006 |
| **Miami** | FL | 25.780 | -80.281 | 1/1/1985 | 12/31/2006 |
| **Middlesex** | NJ | 40.576 | -74.501 | 1/1/1985 | 12/31/2006 |
| **Middletown** | IN | 40.047 | -85.537 | 1/3/1985 | 12/31/2006 |
| **Middletown** | OH | 39.532 | -84.390 | 1/1/1985 | 12/31/2006 |
| **Milwaukee** | WI | 43.069 | -87.964 | 1/1/1985 | 12/31/2006 |
| **Minneapolis** | MN | 44.961 | -93.264 | 1/1/1985 | 12/31/2006 |
| **Missoula** | MT | 46.860 | -114.018 | 1/1/1985 | 12/30/2006 |
| **Mobile** | AL | 30.678 | -88.126 | 1/1/1985 | 12/31/2006 |
| **Modesto** | CA | 37.663 | -120.993 | 1/1/1985 | 12/31/2006 |
| **Monroe** | LA | 32.541 | -92.074 | 1/2/1985 | 12/31/2006 |
| **Monroe** | MI | 41.916 | -83.397 | 1/1/1985 | 12/31/2006 |
| **Montgomery** | AL | 32.360 | -86.273 | 1/1/1985 | 12/31/2006 |
| **Muncie** | IN | 40.198 | -85.394 | 1/1/1985 | 12/31/2006 |
| **Muskegon** | MI | 43.221 | -86.237 | 1/1/1985 | 12/31/2006 |
| **Myrtle Beach** | SC | 33.729 | -78.859 | 1/1/1985 | 12/31/2011 |
| **Nampa** | ID | 43.574 | -116.562 | 1/1/1985 | 12/31/1993 |
| **Naples** | FL | 26.169 | -81.733 | 1/1/1985 | 12/31/2006 |
| **Nashua** | NH | 42.751 | -71.490 | 1/1/1985 | 12/31/2006 |
| **Nashville** | TN | 36.151 | -86.775 | 1/1/1985 | 12/31/2006 |
| **New Haven** | CT | 41.313 | -72.941 | 1/1/1985 | 12/31/2006 |
| **New London** | CT | 41.351 | -72.106 | 1/1/1985 | 12/31/2006 |
| **New Orleans** | LA | 29.976 | -90.047 | 1/1/1985 | 12/31/2006 |
| **New York** | NY | 40.713 | -74.006 | 1/1/1985 | 12/31/2006 |
| **Newark** | NJ | 40.734 | -74.192 | 1/1/1985 | 12/31/2006 |
| **Newburgh** | NY | 41.508 | -74.010 | 1/1/1985 | 12/31/2006 |
| **Niles** | MI | 41.820 | -86.237 | 1/1/1985 | 12/31/2006 |
| **Norfolk** | VA | 36.881 | -76.266 | 1/1/1985 | 12/31/2006 |
| **Oakland** | CA | 37.790 | -122.218 | 1/1/1985 | 12/31/2006 |
| **Ocala** | FL | 29.187 | -82.140 | 1/1/1985 | 12/31/2006 |
| **Ogden** | UT | 41.232 | -111.969 | 1/1/1985 | 12/31/2006 |
| **Oklahoma City** | OK | 35.480 | -97.530 | 1/1/1985 | 12/31/2006 |
| **Orlando** | FL | 28.492 | -81.344 | 1/1/1985 | 12/31/2006 |
| **Osceola** | MO | 38.029 | -93.754 | 1/1/1985 | 12/23/2006 |
| **Ottawa** | IL | 41.353 | -88.842 | 1/1/1985 | 12/31/2006 |
| **Palm Beach** | FL | 26.721 | -80.039 | 1/1/1985 | 12/31/2006 |
| **Paterson** | NJ | 40.916 | -74.163 | 1/1/1985 | 12/31/2006 |
| **Pell City** | AL | 33.598 | -86.343 | 1/2/1985 | 12/31/2006 |
| **Pensacola** | FL | 30.448 | -87.259 | 1/1/1985 | 12/31/2006 |
| **Peoria** | IL | 40.709 | -89.621 | 1/1/1985 | 12/30/2006 |
| **Philadelphia** | PA | 39.998 | -75.149 | 1/1/1985 | 12/31/2006 |
| **Phoenix** | AZ | 33.505 | -112.093 | 1/1/1985 | 12/31/2006 |
| **Pittsburgh** | PA | 40.441 | -79.996 | 1/1/1985 | 12/31/2006 |
| **Plymouth** | MA | 41.910 | -70.642 | 1/1/1985 | 12/31/2013 |
| **Port Arthur** | TX | 29.921 | -93.927 | 1/1/1985 | 12/31/2006 |
| **Portage** | IN | 41.567 | -87.176 | 1/1/1985 | 12/31/2006 |
| **Portland** | ME | 43.669 | -70.279 | 1/1/1985 | 12/31/2006 |
| **Portland** | OR | 45.527 | -122.650 | 1/1/1985 | 12/31/2006 |
| **Portsmouth** | NH | 43.071 | -70.762 | 1/1/1985 | 12/31/2006 |
| **Providence** | RI | 41.831 | -71.417 | 1/1/1985 | 12/31/2010 |
| **Provo** | UT | 40.242 | -111.658 | 1/1/1985 | 12/31/2006 |
| **Punta Gorda** | FL | 26.928 | -81.995 | 1/1/1985 | 12/31/2006 |
| **Raleigh** | NC | 35.821 | -78.652 | 1/1/1985 | 12/31/2006 |
| **Reading** | PA | 40.348 | -75.929 | 1/1/1985 | 12/31/2006 |
| **Reno** | NV | 39.551 | -119.799 | 1/1/1985 | 12/31/2006 |
| **Richmond** | VA | 37.531 | -77.471 | 1/1/1985 | 12/31/2006 |
| **Riverside** | CA | 33.940 | -117.392 | 1/1/1985 | 12/31/2006 |
| **Rochester** | MN | 44.021 | -92.454 | 1/1/1985 | 12/31/2006 |
| **Rochester** | NY | 43.171 | -77.620 | 1/1/1985 | 12/31/2006 |
| **Rockford** | IL | 42.262 | -89.060 | 1/1/1985 | 12/31/2006 |
| **Rockville** | MD | 39.075 | -77.127 | 1/1/1985 | 12/31/2006 |
| **Sacramento** | CA | 38.575 | -121.438 | 1/1/1985 | 12/31/2006 |
| **Saginaw** | MI | 43.433 | -83.961 | 1/1/1985 | 12/31/2006 |
| **Salem** | MA | 42.515 | -70.900 | 1/1/1985 | 12/31/2013 |
| **Salinas** | CA | 36.665 | -121.648 | 1/1/1985 | 12/31/2006 |
| **Salt Lake City** | UT | 40.749 | -111.866 | 1/1/1985 | 12/31/2006 |
| **San Antonio** | TX | 29.466 | -98.529 | 1/1/1985 | 12/31/2006 |
| **San Diego** | CA | 32.793 | -117.136 | 1/1/1985 | 12/31/2006 |
| **San Francisco** | CA | 37.769 | -122.430 | 1/1/1985 | 12/31/2006 |
| **San Jose** | CA | 37.303 | -121.863 | 1/1/1985 | 12/31/2006 |
| **Santa Barbara** | CA | 34.429 | -119.734 | 1/1/1985 | 12/31/2006 |
| **Santa Rosa** | CA | 38.447 | -122.705 | 1/1/1985 | 12/31/2006 |
| **Sarasota** | FL | 27.331 | -82.485 | 1/1/1985 | 12/31/2006 |
| **Savannah** | GA | 32.007 | -81.084 | 1/1/1985 | 12/31/2011 |
| **Scranton** | PA | 41.415 | -75.662 | 1/1/1985 | 12/31/2006 |
| **Seattle** | WA | 47.630 | -122.340 | 1/1/1985 | 12/31/2006 |
| **Shreveport** | LA | 32.461 | -93.789 | 1/1/1985 | 12/31/2006 |
| **Sioux City** | IA | 42.486 | -96.393 | 1/1/1985 | 12/31/2006 |
| **South Bend** | IN | 41.682 | -86.251 | 1/1/1985 | 12/31/2006 |
| **Spartanburg** | SC | 34.946 | -81.920 | 1/1/1985 | 12/31/2011 |
| **Spokane** | WA | 47.673 | -117.375 | 1/1/1985 | 12/31/2006 |
| **Spring Hill** | FL | 28.482 | -82.570 | 1/1/1985 | 12/31/2006 |
| **Springfield** | IL | 39.800 | -89.650 | 1/1/1985 | 12/31/2006 |
| **Springfield** | MA | 42.108 | -72.548 | 1/1/1985 | 12/31/2013 |
| **Springfield** | MO | 37.186 | -93.278 | 1/1/1985 | 12/31/2006 |
| **St. Charles** | MO | 38.767 | -90.559 | 1/1/1985 | 12/31/2006 |
| **St. Louis** | MO | 38.637 | -90.244 | 1/1/1985 | 12/31/2006 |
| **Saint Petersburg** | FL | 27.781 | -82.666 | 1/1/1985 | 12/31/2006 |
| **Stamford** | CT | 41.081 | -73.538 | 1/1/1985 | 12/31/2006 |
| **State College** | PA | 40.800 | -77.872 | 1/1/1985 | 12/31/2006 |
| **Ste. Genevieve** | MO | 37.978 | -90.047 | 1/1/1985 | 5/25/1985 |
| **Steubenville** | OH | 40.370 | -80.623 | 1/1/1985 | 12/31/2006 |
| **Stockton** | CA | 37.987 | -121.295 | 1/1/1985 | 12/31/2006 |
| **Syracuse** | NY | 43.044 | -76.149 | 1/1/1985 | 12/31/2006 |
| **Tacoma** | WA | 47.243 | -122.457 | 1/1/1985 | 12/31/2006 |
| **Tallahassee** | FL | 30.456 | -84.273 | 1/2/1985 | 12/30/2006 |
| **Tampa** | FL | 27.993 | -82.478 | 1/1/1985 | 12/31/2006 |
| **Tavares** | FL | 28.801 | -81.734 | 1/1/1985 | 12/31/2006 |
| **Terre Haute** | IN | 39.459 | -87.388 | 1/1/1985 | 12/31/2006 |
| **Toledo** | OH | 41.667 | -83.586 | 1/1/1985 | 12/31/2006 |
| **Toms River** | NJ | 39.983 | -74.210 | 1/1/1985 | 12/31/2006 |
| **Topeka** | KS | 39.048 | -95.693 | 1/1/1985 | 12/31/2006 |
| **Trenton** | NJ | 40.236 | -74.769 | 1/1/1985 | 12/31/2006 |
| **Tucson** | AZ | 32.194 | -110.949 | 1/1/1985 | 12/31/2006 |
| **Tulsa** | OK | 36.135 | -95.935 | 1/1/1985 | 12/31/2006 |
| **Tyler** | TX | 32.346 | -95.295 | 1/1/1985 | 12/28/2006 |
| **Greater Upper Marlboro** | MD | 38.838 | -76.798 | 1/1/1985 | 12/31/2006 |
| **Utica** | NY | 43.097 | -75.231 | 1/1/1985 | 9/23/1998 |
| **Vancouver** | WA | 45.656 | -122.616 | 1/1/1985 | 12/31/2006 |
| **Ventura** | CA | 34.280 | -119.226 | 1/1/1985 | 12/31/2006 |
| **Visalia** | CA | 36.333 | -119.304 | 1/1/1985 | 12/31/2006 |
| **Washington** | DC | 38.909 | -77.022 | 1/1/1985 | 12/31/2006 |
| **Washington** | PA | 40.172 | -80.256 | 1/1/1985 | 12/31/2006 |
| **West Orange** | TX | 30.125 | -93.772 | 1/2/1985 | 12/31/2006 |
| **Wichita** | KS | 37.665 | -97.337 | 1/1/1985 | 12/31/2006 |
| **Wilmington** | DE | 39.749 | -75.557 | 1/1/1985 | 12/31/2006 |
| **Winston-Salem** | NC | 36.090 | -80.264 | 1/1/1985 | 12/31/2006 |
| **Woodstock** | IL | 42.320 | -88.448 | 1/1/1985 | 12/31/2006 |
| **Worcester** | MA | 42.265 | -71.807 | 1/1/1985 | 12/31/2006 |
| **Yonkers** | NY | 40.939 | -73.874 | 1/1/1985 | 12/31/2006 |
| **Youngstown** | OH | 41.099 | -80.657 | 1/1/1985 | 12/31/2006 |
| **Gulfport** | MS | 30.413 | -89.074 | 1/2/1985 | 12/31/2006 |
| **New Braunfels** | TX | 29.703 | -98.124 | 1/2/1985 | 12/29/2006 |
| **Carlisle** | PA | 40.201 | -77.200 | 6/1/1985 | 12/31/2007 |
| **Harrisburg** | PA | 40.286 | -76.862 | 6/1/1985 | 12/30/2005 |
| **Lancaster** | PA | 39.956 | -76.720 | 6/1/1985 | 12/30/2005 |
| **York** | PA | 39.956 | -76.720 | 6/1/1985 | 12/30/2005 |

Table S3. City-recorded deaths

|  |  |  |  | **City-recorded deaths** | | | | |
| --- | --- | --- | --- | --- | --- | --- | --- | --- |
| **CITY_NAME** | **State** | **Latitude** | **Longitude** | **TOTAL** | **CVD** | **MI** | **STROKE** | **CHF** |
| **Akron** | OH | 41.068 | -81.522 | 107392 | 34190 | 9703 | 6672 | 3520 |
| **Albany** | NY | 42.658 | -73.767 | 67639 | 25818 | 4746 | 3947 | 1631 |
| **Albuquerque** | NM | 35.103 | -106.602 | 85958 | 23442 | 4130 | 5437 | 1955 |
| **Allentown** | PA | 40.600 | -75.484 | 69778 | 24147 | 7314 | 4039 | 1571 |
| **Alpine** | TX | 30.263 | -103.654 | 1155 | 339 | 137 | 94 | 51 |
| **Anaheim** | CA | 33.836 | -117.881 | 351872 | 120198 | 32023 | 24267 | 4599 |
| **Ann Arbor** | MI | 42.276 | -83.727 | 39006 | 12144 | 3070 | 2488 | 920 |
| **Annandale** | VA | 38.831 | -77.214 | 58855 | 16835 | 3461 | 3942 | 1761 |
| **Annapolis** | MD | 38.952 | -76.491 | 73158 | 22153 | 5748 | 4292 | 1525 |
| **Asheville** | NC | 35.591 | -82.548 | 47444 | 14401 | 4154 | 3699 | 1139 |
| **Ashland** | KY | 38.461 | -82.645 | 9157 | 2994 | 1222 | 812 | 352 |
| **Athens** | GA | 33.965 | -83.390 | 10676 | 3279 | 691 | 767 | 222 |
| **Atlanta** | GA | 33.795 | -84.387 | 12537216 | 3628832 | 695296 | 811264 | 321760 |
| **Atlantic City** | NJ | 39.366 | -74.432 | 53725 | 18870 | 4935 | 3112 | 720 |
| **Augusta** | GA | 33.467 | -81.993 | 45426 | 13972 | 4550 | 2784 | 840 |
| **Aurora** | CO | 39.696 | -104.791 | 41304 | 11130 | 3139 | 2495 | 906 |
| **Austin** | TX | 30.308 | -97.763 | 1340224 | 403040 | 109184 | 100544 | 25920 |
| **Aztec** | NM | 36.820 | -108.011 | 9576 | 2529 | 521 | 423 | 239 |
| **Bakersfield** | CA | 35.351 | -119.028 | 98359 | 35196 | 8317 | 5737 | 1588 |
| **Baltimore** | MD | 39.312 | -76.619 | 357964 | 114008 | 30682 | 21393 | 6094 |
| **Bangor** | ME | 44.824 | -68.792 | 30317 | 9689 | 2893 | 1944 | 770 |
| **Bath** | NY | 42.337 | -77.318 | 13756 | 4702 | 1473 | 812 | 333 |
| **Baton Rouge** | LA | 30.444 | -91.098 | 27154 | 8913 | 2889 | 1693 | 1383 |
| **Beaver Dam** | WI | 43.462 | -88.841 | 11259 | 4200 | 1525 | 999 | 342 |
| **Bel Air** | MD | 39.535 | -76.336 | 31611 | 10626 | 2793 | 1775 | 538 |
| **Bend** | OR | 44.058 | -121.296 | 13328 | 3671 | 842 | 972 | 284 |
| **Birmingham** | AL | 33.523 | -86.826 | 171109 | 53380 | 13326 | 12880 | 6179 |
| **Boise** | ID | 43.611 | -116.232 | 21174 | 6769 | 2196 | 1510 | 406 |
| **Boston** | MA | 42.354 | -71.069 | 619833 | 199359 | 34581 | 28472 | 8178 |
| **Boulder** | CO | 40.024 | -105.254 | 22324 | 6380 | 1239 | 1620 | 531 |
| **Brownsville** | TX | 25.928 | -97.489 | 42532 | 12909 | 4859 | 2646 | 721 |
| **Burlington** | VT | 44.484 | -73.220 | 19873 | 6319 | 1414 | 1112 | 335 |
| **Canton** | OH | 40.813 | -81.377 | 77288 | 26330 | 7977 | 5269 | 1631 |
| **Cedar Rapids** | IA | 41.982 | -91.668 | 28113 | 8505 | 2093 | 2014 | 298 |
| **Charleston** | SC | 32.835 | -80.010 | 62488 | 17881 | 4074 | 4931 | 1246 |
| **Charleston** | WV | 38.350 | -81.633 | 49105 | 16916 | 4320 | 2912 | 1233 |
| **Charlotte** | NC | 35.202 | -80.832 | 95918 | 26882 | 6713 | 7010 | 1846 |
| **Chattanooga** | TN | 35.036 | -85.277 | 60219 | 20939 | 5661 | 4443 | 791 |
| **Chesterbrook** | PA | 40.075 | -75.459 | 69158 | 22537 | 5702 | 4414 | 1770 |
| **Chesterfield** | SC | 34.728 | -80.096 | 6692 | 2127 | 956 | 497 | 120 |
| **Chicago** | IL | 41.872 | -87.669 | 1115158 | 392344 | 117331 | 70033 | 21839 |
| **Chico** | CA | 39.730 | -121.816 | 42983 | 12824 | 2943 | 3275 | 937 |
| **Cincinnati** | OH | 39.144 | -84.512 | 171912 | 56901 | 10856 | 11070 | 3491 |
| **Cleveland** | OH | 41.489 | -81.652 | 404057 | 149729 | 30280 | 23582 | 8537 |
| **Colorado Springs** | CO | 38.846 | -104.741 | 60542 | 15133 | 3455 | 4876 | 815 |
| **Columbia** | SC | 34.031 | -81.018 | 97116 | 28145 | 9990 | 6906 | 1954 |
| **Columbus** | GA | 32.482 | -84.947 | 42369 | 13912 | 3219 | 2687 | 1053 |
| **Columbus** | OH | 39.981 | -82.989 | 159353 | 49659 | 13167 | 9534 | 3272 |
| **Conroe** | TX | 30.242 | -95.458 | 39920 | 12452 | 3310 | 2197 | 1474 |
| **Corpus Christi** | TX | 27.756 | -97.414 | 54740 | 16131 | 4124 | 3646 | 1072 |
| **Covington** | KY | 39.071 | -84.521 | 27757 | 8952 | 2705 | 1588 | 675 |
| **Dade City** | FL | 28.364 | -82.195 | 158555 | 52300 | 11627 | 10457 | 1145 |
| **Dallas** | TX | 32.801 | -96.791 | 301424 | 96645 | 20338 | 20410 | 5480 |
| **Davenport** | IA | 41.546 | -90.586 | 55256 | 19131 | 6615 | 4563 | 1595 |
| **Dayton** | OH | 39.762 | -84.197 | 108776 | 36036 | 9552 | 7169 | 1900 |
| **Daytona Beach** | FL | 29.212 | -81.023 | 107272 | 37076 | 9145 | 7331 | 1065 |
| **Denver** | CO | 39.718 | -104.962 | 165320 | 45874 | 12243 | 9557 | 3242 |
| **Des Moines** | IA | 41.588 | -93.632 | 54488 | 18548 | 3593 | 3299 | 698 |
| **Detroit** | MI | 42.368 | -83.106 | 826492 | 307724 | 80133 | 48542 | 17343 |
| **Dover** | DE | 39.157 | -75.536 | 14844 | 5418 | 1788 | 954 | 313 |
| **Duluth** | MN | 46.822 | -92.081 | 50498 | 16011 | 3547 | 3598 | 1673 |
| **Durham** | NC | 36.013 | -78.909 | 36955 | 10270 | 2591 | 2561 | 900 |
| **Dutchess** | NY | 41.778 | -73.747 | 40780 | 15015 | 4690 | 2310 | 790 |
| **East Saint Louis** | IL | 38.599 | -90.074 | 48907 | 16740 | 5643 | 3526 | 1426 |
| **El Centro** | CA | 32.789 | -115.567 | 17575 | 5586 | 1972 | 1274 | 246 |
| **El Paso** | TX | 31.805 | -106.435 | 85341 | 24789 | 5388 | 5316 | 1359 |
| **Elizabeth** | NJ | 40.664 | -74.211 | 104912 | 36530 | 11227 | 6315 | 1895 |
| **Elkhart** | IN | 41.678 | -85.969 | 29048 | 10175 | 3161 | 2266 | 850 |
| **Erie** | PA | 42.108 | -80.081 | 62246 | 21260 | 6956 | 4787 | 1531 |
| **Eugene** | OR | 44.055 | -123.113 | 61134 | 16259 | 3680 | 4632 | 1605 |
| **Evansville** | IN | 37.993 | -87.553 | 40894 | 13586 | 4532 | 3021 | 1378 |
| **Everett** | WA | 47.943 | -122.197 | 76017 | 22672 | 4773 | 5537 | 1146 |
| **Fargo** | ND | 46.856 | -96.812 | 16438 | 5052 | 1522 | 1256 | 397 |
| **Fayetteville** | NC | 35.054 | -78.944 | 31267 | 9955 | 2454 | 2029 | 462 |
| **Flint** | MI | 43.015 | -83.692 | 86502 | 30899 | 9547 | 5882 | 2609 |
| **Fort Lauderdale** | FL | 26.120 | -80.199 | 308032 | 115757 | 33179 | 20139 | 3016 |
| **Fort Myers** | FL | 26.593 | -81.852 | 88850 | 32406 | 5922 | 5267 | 669 |
| **Fort Pierce** | FL | 27.445 | -80.357 | 67004 | 23799 | 4979 | 4240 | 462 |
| **Fort Wayne** | IN | 41.084 | -85.138 | 55758 | 18521 | 4735 | 4137 | 1982 |
| **Fort Worth** | TX | 32.746 | -97.318 | 203286 | 64980 | 19497 | 14360 | 3939 |
| **Fredericktown** | MO | 37.559 | -90.294 | 72 | 28 | 10 | 4 | 1 |
| **Fresno** | CA | 36.783 | -119.786 | 104033 | 34869 | 9636 | 7489 | 2095 |
| **Gainesville** | FL | 29.659 | -82.360 | 27589 | 7034 | 2029 | 2092 | 333 |
| **Galveston** | TX | 29.268 | -94.846 | 17932 | 6110 | 1790 | 1081 | 392 |
| **Gary** | IN | 41.578 | -87.343 | 90669 | 32264 | 7661 | 6341 | 2552 |
| **Geneva** | IL | 41.886 | -88.311 | 48008 | 15873 | 4486 | 3364 | 1039 |
| **Gettysburg** | PA | 39.832 | -77.222 | 10597 | 4081 | 1143 | 684 | 315 |
| **Grand Haven** | MI | 43.038 | -86.191 | 26506 | 8609 | 2424 | 1937 | 1027 |
| **Grand Junction** | CO | 39.086 | -108.567 | 10047 | 3176 | 871 | 621 | 178 |
| **Grand Rapids** | MI | 42.956 | -85.621 | 78804 | 26402 | 5413 | 5335 | 1754 |
| **Green Bay** | WI | 44.513 | -88.002 | 29880 | 10391 | 3170 | 2469 | 1170 |
| **Greensboro** | NC | 36.075 | -79.826 | 65906 | 19847 | 6166 | 5651 | 1079 |
| **Greensburg** | PA | 40.307 | -79.542 | 86732 | 33103 | 10591 | 5558 | 2112 |
| **Greenville** | SC | 34.848 | -82.387 | 74574 | 22579 | 6630 | 5272 | 1628 |
| **Hartford** | CT | 41.767 | -72.687 | 159050 | 55065 | 11970 | 9651 | 3158 |
| **Hickory** | NC | 35.723 | -81.345 | 23169 | 7196 | 2815 | 1794 | 528 |
| **Holland** | MI | 42.791 | -86.129 | 10159 | 3456 | 908 | 646 | 365 |
| **Houston** | TX | 29.776 | -95.416 | 366340 | 117245 | 30163 | 25662 | 7942 |
| **Huntsville** | AL | 34.711 | -86.613 | 37568 | 13120 | 3674 | 2445 | 2388 |
| **Indianapolis** | IN | 39.789 | -86.141 | 149459 | 46702 | 10661 | 9517 | 4552 |
| **Iowa City** | IA | 41.655 | -91.531 | 6600 | 1977 | 621 | 510 | 83 |
| **Ironton** | OH | 38.529 | -82.665 | 7235 | 2802 | 1171 | 367 | 260 |
| **Jackson** | MS | 32.318 | -90.198 | 44055 | 16502 | 4483 | 2964 | 1290 |
| **Jacksonville** | FL | 30.295 | -81.653 | 124017 | 39254 | 9270 | 8405 | 1174 |
| **Jersey City** | NJ | 40.726 | -74.062 | 103084 | 36328 | 9733 | 5463 | 2007 |
| **Joliet** | IL | 41.526 | -88.065 | 54496 | 19344 | 6712 | 3482 | 1240 |
| **Kalamazoo** | MI | 42.264 | -85.610 | 35666 | 11387 | 2993 | 2401 | 1200 |
| **Kansas City** | KS | 39.111 | -94.685 | 218933 | 69986 | 18207 | 14770 | 6025 |
| **Kenosha** | WI | 42.582 | -87.852 | 23194 | 8165 | 1907 | 1665 | 640 |
| **Klamath Falls** | OR | 42.192 | -121.724 | 8149 | 2345 | 780 | 599 | 154 |
| **Knoxville** | TN | 35.990 | -83.953 | 80418 | 23605 | 7589 | 5904 | 1050 |
| **La Porte** | IN | 41.599 | -86.708 | 20183 | 7275 | 2228 | 1313 | 442 |
| **Lafayette** | IN | 40.415 | -86.874 | 19480 | 6356 | 1863 | 1511 | 430 |
| **Lafayette** | LA | 30.214 | -92.033 | 24497 | 7478 | 1892 | 1603 | 681 |
| **Lake Charles** | LA | 30.240 | -93.207 | 31476 | 11398 | 1784 | 1776 | 887 |
| **Lake City** | MI | 44.335 | -85.215 | 1667 | 624 | 211 | 125 | 42 |
| **Lakeland** | FL | 28.062 | -81.959 | 95395 | 34558 | 8089 | 5954 | 794 |
| **Lansing** | MI | 42.727 | -84.566 | 37393 | 12471 | 3082 | 2595 | 1501 |
| **Las Vegas** | NV | 36.148 | -115.174 | 177062 | 56530 | 9751 | 10118 | 4564 |
| **Lawrenceburg** | TN | 35.251 | -87.353 | 5341 | 1928 | 952 | 358 | 88 |
| **Layton** | UT | 41.086 | -111.949 | 12685 | 3859 | 841 | 947 | 407 |
| **Lexington** | KY | 38.028 | -84.499 | 36826 | 11379 | 3596 | 2392 | 934 |
| **Lisbon** | OH | 40.759 | -80.759 | 89751 | 32440 | 12376 | 5864 | 2213 |
| **Little Rock** | AR | 34.734 | -92.355 | 48163 | 14565 | 4623 | 4318 | 1381 |
| **Logan** | UT | 41.747 | -111.823 | 4959 | 1445 | 412 | 404 | 169 |
| **Los Angeles** | CA | 34.042 | -118.305 | 1239036 | 460300 | 110590 | 85968 | 14071 |
| **Louisville** | KY | 38.223 | -85.731 | 139347 | 46751 | 14092 | 9214 | 4345 |
| **Lubbock** | TX | 33.564 | -101.881 | 35407 | 12154 | 3071 | 2333 | 1055 |
| **Macon** | GA | 32.874 | -83.675 | 40383 | 12920 | 3485 | 3051 | 1101 |
| **Madison** | IL | 38.681 | -90.157 | 48230 | 18039 | 6685 | 3296 | 1878 |
| **Marshall** | TX | 32.534 | -94.362 | 7476 | 2500 | 1028 | 694 | 124 |
| **McAllen** | TX | 26.216 | -98.239 | 49922 | 16560 | 7325 | 2895 | 874 |
| **Medford** | OR | 42.336 | -122.840 | 33409 | 9526 | 2034 | 3016 | 834 |
| **Medford** | WI | 45.151 | -90.350 | 2387 | 839 | 329 | 173 | 63 |
| **Melbourne** | FL | 28.138 | -80.652 | 63243 | 21602 | 6311 | 3929 | 515 |
| **Melville** | NY | 40.795 | -73.403 | 460192 | 187765 | 40099 | 22866 | 7728 |
| **Memphis** | TN | 35.122 | -89.960 | 152003 | 52592 | 12004 | 12578 | 1947 |
| **Miami** | FL | 25.780 | -80.281 | 372130 | 139188 | 37659 | 21561 | 2995 |
| **Middlesex** | NJ | 40.576 | -74.501 | 110324 | 37784 | 12049 | 5782 | 1913 |
| **Middletown** | IN | 40.047 | -85.537 | 7312 | 2409 | 966 | 713 | 250 |
| **Middletown** | OH | 39.532 | -84.390 | 1587776 | 503808 | 149344 | 100128 | 34944 |
| **Milwaukee** | WI | 43.069 | -87.964 | 232056 | 78803 | 18860 | 16907 | 5001 |
| **Minneapolis** | MN | 44.961 | -93.264 | 241475 | 64293 | 13722 | 18812 | 6274 |
| **Missoula** | MT | 46.860 | -114.018 | 7826 | 2093 | 504 | 504 | 240 |
| **Mobile** | AL | 30.678 | -88.126 | 72746 | 23649 | 5877 | 4717 | 1896 |
| **Modesto** | CA | 37.663 | -120.993 | 63548 | 21576 | 6082 | 4482 | 1028 |
| **Monroe** | LA | 32.541 | -92.074 | 26413 | 8736 | 1776 | 1686 | 734 |
| **Monroe** | MI | 41.916 | -83.397 | 18590 | 7006 | 1575 | 1000 | 376 |
| **Montgomery** | AL | 32.360 | -86.273 | 40166 | 12567 | 2970 | 2998 | 1220 |
| **Muncie** | IN | 40.198 | -85.394 | 23145 | 7882 | 2106 | 1649 | 538 |
| **Muskegon** | MI | 43.221 | -86.237 | 30902 | 10858 | 3133 | 2182 | 552 |
| **Myrtle Beach** | SC | 33.729 | -78.859 | 41244 | 12466 | 4774 | 2946 | 793 |
| **Nampa** | ID | 43.574 | -116.562 | 6199 | 2170 | 764 | 581 | 173 |
| **Naples** | FL | 26.169 | -81.733 | 36806 | 11706 | 2883 | 2248 | 301 |
| **Nashua** | NH | 42.751 | -71.490 | 51115 | 17169 | 3942 | 3265 | 750 |
| **Nashville** | TN | 36.151 | -86.775 | 97358 | 32531 | 9295 | 7111 | 1358 |
| **New Haven** | CT | 41.313 | -72.941 | 157415 | 53827 | 11375 | 9675 | 3079 |
| **New London** | CT | 41.351 | -72.106 | 40419 | 13406 | 2970 | 2850 | 1165 |
| **New Orleans** | LA | 29.976 | -90.047 | 180593 | 56324 | 16643 | 12387 | 4164 |
| **New York** | NY | 40.713 | -74.006 | 1367085 | 598000 | 141476 | 50972 | 15551 |
| **Newark** | NJ | 40.734 | -74.192 | 220980 | 72454 | 24171 | 12840 | 4042 |
| **Newburgh** | NY | 41.508 | -74.010 | 40562 | 15419 | 4854 | 2217 | 836 |
| **Niles** | MI | 41.820 | -86.237 | 30355 | 10232 | 2990 | 2438 | 641 |
| **Norfolk** | VA | 36.881 | -76.266 | 187233 | 59837 | 14356 | 12974 | 4968 |
| **Oakland** | CA | 37.790 | -122.218 | 325028 | 102757 | 24282 | 25557 | 4485 |
| **Ocala** | FL | 29.187 | -82.140 | 58345 | 20284 | 4987 | 4579 | 404 |
| **Ogden** | UT | 41.232 | -111.969 | 18088 | 5793 | 1498 | 1353 | 670 |
| **Oklahoma City** | OK | 35.480 | -97.530 | 118753 | 40329 | 8141 | 8975 | 4344 |
| **Orlando** | FL | 28.492 | -81.344 | 157019 | 51708 | 13336 | 9460 | 1742 |
| **Osceola** | MO | 38.029 | -93.754 | 1824 | 698 | 249 | 129 | 78 |
| **Ottawa** | IL | 41.353 | -88.842 | 25070 | 9313 | 3338 | 1769 | 494 |
| **Palm Beach** | FL | 26.721 | -80.039 | 233887 | 86882 | 20794 | 15476 | 1876 |
| **Paterson** | NJ | 40.916 | -74.163 | 239023 | 87628 | 26983 | 14766 | 4410 |
| **Pell City** | AL | 33.598 | -86.343 | 7333 | 2513 | 908 | 462 | 434 |
| **Pensacola** | FL | 30.448 | -87.259 | 50143 | 15766 | 4081 | 3898 | 378 |
| **Peoria** | IL | 40.709 | -89.621 | 36108 | 11194 | 4143 | 3545 | 1191 |
| **Philadelphia** | PA | 39.998 | -75.149 | 911888 | 299923 | 85819 | 60124 | 18729 |
| **Phoenix** | AZ | 33.505 | -112.093 | 386464 | 121382 | 31314 | 24351 | 4689 |
| **Pittsburgh** | PA | 40.441 | -79.996 | 317935 | 115426 | 32996 | 20399 | 7803 |
| **Plymouth** | MA | 41.910 | -70.642 | 105871 | 35538 | 4434 | 4351 | 1724 |
| **Port Arthur** | TX | 29.921 | -93.927 | 50961 | 17792 | 5186 | 3818 | 1551 |
| **Portage** | IN | 41.567 | -87.176 | 20147 | 6800 | 1622 | 1338 | 589 |
| **Portland** | ME | 43.669 | -70.279 | 46217 | 14002 | 3523 | 2971 | 1180 |
| **Portland** | OR | 45.527 | -122.650 | 210301 | 60902 | 14201 | 17690 | 5077 |
| **Portsmouth** | NH | 43.071 | -70.762 | 30362 | 9641 | 2469 | 1991 | 480 |
| **Providence** | RI | 41.831 | -71.417 | 281243 | 98562 | 31715 | 17373 | 4557 |
| **Provo** | UT | 40.242 | -111.658 | 27214 | 8334 | 2005 | 1992 | 1288 |
| **Punta Gorda** | FL | 26.928 | -81.995 | 37641 | 13718 | 3519 | 2229 | 457 |
| **Raleigh** | NC | 35.821 | -78.652 | 58561 | 17337 | 4368 | 4851 | 1019 |
| **Reading** | PA | 40.348 | -75.929 | 72308 | 25615 | 8181 | 5287 | 1561 |
| **Reno** | NV | 39.551 | -119.799 | 45340 | 15056 | 3037 | 2504 | 988 |
| **Richmond** | VA | 37.531 | -77.471 | 116646 | 35624 | 8497 | 8939 | 2910 |
| **Riverside** | CA | 33.940 | -117.392 | 433172 | 152979 | 38156 | 28543 | 5939 |
| **Rochester** | MN | 44.021 | -92.454 | 14413 | 4130 | 1347 | 1031 | 242 |
| **Rochester** | NY | 43.171 | -77.620 | 127040 | 41465 | 17644 | 8465 | 3130 |
| **Rockford** | IL | 42.262 | -89.060 | 46380 | 15355 | 4186 | 3365 | 1370 |
| **Rockville** | MD | 39.075 | -77.127 | 92381 | 28624 | 7851 | 6734 | 1618 |
| **Sacramento** | CA | 38.575 | -121.438 | 172136 | 54279 | 13054 | 12393 | 2397 |
| **Saginaw** | MI | 43.433 | -83.961 | 39485 | 14023 | 3980 | 2696 | 1158 |
| **Salem** | MA | 42.515 | -70.900 | 175164 | 60502 | 10182 | 8163 | 2119 |
| **Salinas** | CA | 36.665 | -121.648 | 45929 | 13973 | 3076 | 3770 | 583 |
| **Salt Lake City** | UT | 40.749 | -111.866 | 89770 | 25224 | 5923 | 6146 | 3033 |
| **San Antonio** | TX | 29.466 | -98.529 | 186461 | 61295 | 16658 | 12295 | 3282 |
| **San Diego** | CA | 32.793 | -117.136 | 369956 | 118268 | 31542 | 26480 | 5288 |
| **San Francisco** | CA | 37.769 | -122.430 | 248607 | 76041 | 19152 | 20148 | 3208 |
| **San Jose** | CA | 37.303 | -121.863 | 127752 | 41304 | 10347 | 9694 | 1490 |
| **Santa Barbara** | CA | 34.429 | -119.734 | 56600 | 18852 | 4055 | 4621 | 867 |
| **Santa Rosa** | CA | 38.447 | -122.705 | 72662 | 22043 | 4988 | 6198 | 999 |
| **Sarasota** | FL | 27.331 | -82.485 | 151551 | 52666 | 14086 | 11334 | 1302 |
| **Savannah** | GA | 32.007 | -81.084 | 53125 | 16842 | 4414 | 3575 | 1051 |
| **Scranton** | PA | 41.415 | -75.662 | 150018 | 61754 | 18304 | 8469 | 3392 |
| **Seattle** | WA | 47.630 | -122.340 | 225451 | 66208 | 15328 | 17811 | 3733 |
| **Shreveport** | LA | 32.461 | -93.789 | 51716 | 16816 | 4168 | 3148 | 1112 |
| **Sioux City** | IA | 42.486 | -96.393 | 12986 | 4566 | 1287 | 907 | 239 |
| **South Bend** | IN | 41.682 | -86.251 | 48505 | 16436 | 5297 | 3832 | 1262 |
| **Spartanburg** | SC | 34.946 | -81.920 | 58209 | 18394 | 5614 | 4522 | 1499 |
| **Spokane** | WA | 47.673 | -117.375 | 68681 | 21024 | 5482 | 5421 | 985 |
| **Spring Hill** | FL | 28.482 | -82.570 | 158555 | 52300 | 11627 | 10457 | 1145 |
| **Springfield** | IL | 39.800 | -89.650 | 35671 | 11880 | 3057 | 2583 | 941 |
| **Springfield** | MA | 42.108 | -72.548 | 124917 | 41990 | 6413 | 6066 | 1931 |
| **Springfield** | MO | 37.186 | -93.278 | 43239 | 14306 | 4699 | 3504 | 1177 |
| **St. Charles** | MO | 38.767 | -90.559 | 28225 | 9504 | 2576 | 1655 | 485 |
| **St. Louis** | MO | 38.637 | -90.244 | 312923 | 114576 | 35848 | 20829 | 5302 |
| **Saint Petersburg** | FL | 27.781 | -82.666 | 158555 | 52300 | 11627 | 10457 | 1145 |
| **Stamford** | CT | 41.081 | -73.538 | 142216 | 50912 | 10544 | 9244 | 3236 |
| **State College** | PA | 40.800 | -77.872 | 16205 | 5781 | 1926 | 1132 | 534 |
| **Ste. Genevieve** | MO | 37.978 | -90.047 | 54 | 25 | 8 | 1 | 0 |
| **Steubenville** | OH | 40.370 | -80.623 | 21002 | 8373 | 2408 | 1256 | 564 |
| **Stockton** | CA | 37.987 | -121.295 | 82168 | 27742 | 6160 | 6377 | 1342 |
| **Syracuse** | NY | 43.044 | -76.149 | 84451 | 27235 | 6622 | 6165 | 1479 |
| **Tacoma** | WA | 47.243 | -122.457 | 96086 | 30186 | 6539 | 7401 | 1245 |
| **Tallahassee** | FL | 30.456 | -84.273 | 25858 | 6981 | 1963 | 1920 | 257 |
| **Tampa** | FL | 27.993 | -82.478 | 158555 | 52300 | 11627 | 10457 | 1145 |
| **Tavares** | FL | 28.801 | -81.734 | 50466 | 17616 | 5140 | 3583 | 432 |
| **Terre Haute** | IN | 39.459 | -87.388 | 23821 | 8551 | 2536 | 1827 | 654 |
| **Toledo** | OH | 41.667 | -83.586 | 92004 | 34731 | 9718 | 5993 | 1727 |
| **Toms River** | NJ | 39.983 | -74.210 | 177757 | 67061 | 19707 | 10555 | 3504 |
| **Topeka** | KS | 39.048 | -95.693 | 31950 | 10275 | 2168 | 2450 | 1019 |
| **Trenton** | NJ | 40.236 | -74.769 | 45643 | 16439 | 4867 | 2490 | 540 |
| **Tucson** | AZ | 32.194 | -110.949 | 131053 | 40979 | 9187 | 8575 | 3561 |
| **Tulsa** | OK | 36.135 | -95.935 | 95475 | 33533 | 7658 | 6762 | 2319 |
| **Tyler** | TX | 32.346 | -95.295 | 30951 | 10746 | 4331 | 2272 | 764 |
| **Greater Upper Marlboro** | MD | 38.838 | -76.798 | 61169 | 19905 | 4325 | 3629 | 836 |
| **Utica** | NY | 43.097 | -75.231 | 30793 | 12138 | 3923 | 2297 | 636 |
| **Vancouver** | WA | 45.656 | -122.616 | 37914 | 11469 | 2674 | 2748 | 526 |
| **Ventura** | CA | 34.280 | -119.226 | 87556 | 27711 | 6910 | 6148 | 1071 |
| **Visalia** | CA | 36.333 | -119.304 | 50358 | 17517 | 4903 | 3754 | 1187 |
| **Washington** | DC | 38.909 | -77.022 | 141028 | 43235 | 7406 | 7767 | 2093 |
| **Washington** | PA | 40.172 | -80.256 | 49002 | 17594 | 5756 | 3081 | 1241 |
| **West Orange** | TX | 30.125 | -93.772 | 10293 | 3721 | 1118 | 547 | 405 |
| **Wichita** | KS | 37.665 | -97.337 | 68542 | 21692 | 6108 | 4661 | 2800 |
| **Wilmington** | DE | 39.749 | -75.557 | 76254 | 25048 | 5765 | 4394 | 1695 |
| **Winston-Salem** | NC | 36.090 | -80.264 | 52543 | 15763 | 4261 | 4385 | 1139 |
| **Woodstock** | IL | 42.320 | -88.448 | 28299 | 9314 | 2421 | 1973 | 758 |
| **Worcester** | MA | 42.265 | -71.807 | 135709 | 44082 | 13016 | 7841 | 3988 |
| **Yonkers** | NY | 40.939 | -73.874 | 158548 | 62009 | 15356 | 9283 | 2914 |
| **Youngstown** | OH | 41.099 | -80.657 | 86656 | 31607 | 12097 | 5704 | 2138 |
| **Gulfport** | MS | 30.413 | -89.074 | 23458 | 9352 | 2442 | 1243 | 527 |
| **New Braunfels** | TX | 29.703 | -98.124 | 5180 | 1680 | 684 | 407 | 104 |
| **Carlisle** | PA | 40.201 | -77.200 | 1001152 | 353312 | 112320 | 76192 | 28416 |
| **Harrisburg** | PA | 40.286 | -76.862 | 39268 | 14074 | 4236 | 2488 | 1088 |
| **Lancaster** | PA | 39.956 | -76.720 | 62521 | 22023 | 5402 | 4597 | 1937 |
| **York** | PA | 39.956 | -76.720 | 48808 | 17260 | 6304 | 3294 | 1098 |
